# Bcl-xl as the most promising Bcl-2 family member in targeted treatment of chondrosarcoma

**DOI:** 10.1038/s41389-018-0084-0

**Published:** 2018-09-21

**Authors:** Yvonne de Jong, David Monderer, Emeline Brandinelli, Morgane Monchanin, Brendy E. van den Akker, Jolieke G. van Oosterwijk, Jean Yves Blay, Aurélie Dutour, Judith V. M. G. Bovée

**Affiliations:** 1 Department of Pathology, Leiden University Medical Center, Leiden, The Netherlands; 2 Department of Medical Oncology, Centre Léon Berard, Lyon, France

## Abstract

Chondrosarcomas are malignant cartilage tumors showing relative resistance to conventional chemo- and radiotherapy. Previous studies showed that chondrosarcoma cells could be sensitized to chemotherapy by inhibiting the Bcl-2 family members Bcl-2, Bcl-xl and Bcl-w using ABT-737. In this study we explored the specific role of Bcl-2 family members to identify the most important player in chondrosarcoma cell survival and chemo resistance. Immunohistochemistry was performed on tissue microarrays containing 137 conventional chondrosarcomas of different grades. Selective inhibition of Bcl-2 (S55746) or Bcl-xl (WEHI-539 or A-1155463) and the combination with doxorubicin or cisplatin was investigated in a panel of 8 chondrosarcoma cell lines using presto blue viability assays and caspase 3/7 glo apoptosis assays. In addition Bcl-2 and Bcl-xl inhibition was investigated in an orthotopic Swarm Rat Chondrosarcoma (SRC) model. Bcl-2 and Bcl-xl were most abundantly expressed in the primary tumors, and expression increased with increasing histological grade. A subset of chondrosarcoma cell lines was sensitive to selective inhibition of Bcl-xl, and synergy was observed with doxorubicin or cisplatin in 3 out of 8 chondrosarcoma cell lines resulting in apoptosis. Conversely, selective inhibition of Bcl-2 was not effective in chondrosarcoma cell lines and could not sensitize to chemotherapy. In vivo, selective inhibition of Bcl-xl, but not Bcl-2 resulted in a decrease in tumor growth rate, even though no sensitization to doxorubicin was observed. These results suggest that among the Bcl-2 family members, Bcl-xl is most important for chondrosarcoma survival. Further research is needed to validate whether single or combination treatment with chemotherapy will be beneficial for chondrosarcoma patients.

## Introduction

Chondrosarcomas are malignant primary bone tumors characterized by the production of a hyaline cartilage matrix, with poor vascularization^1^. Histologically chondrosarcomas can be subdivided into conventional, dedifferentiated, mesenchymal, clear cell and periosteal chondrosarcoma. Conventional chondrosarcoma is the most frequent subtype found in 85% of the cases and can either be found in the medulla of the bone (central subtype) or at the surface of the bone (peripheral subtype). The 10-year survival rate for patients with conventional chondrosarcoma depends on the histological grade. Atypical cartilage tumors (previously grade I) show a 10-year survival of 83%, patients with grade II chondrosarcomas show 64% survival and patients with grade III tumors show a 10-year survival rate of 29%^1^. Chondrosarcomas show relative resistance to conventional chemo- and radiotherapy leaving surgery as the only treatment option. As such the 10-year survival rate of chondrosarcoma patients has remained unchanged for the last four decades^2^. Therefore, new treatment options are urgently needed, especially for patients with inoperable or metastatic disease. Apoptosis is a form of programmed cell death, eliminating damaged or unnecessary cells from the body. The intrinsic apoptosis pathway is regulated by B-cell lymphoma-2 (Bcl-2) family of proteins that comprises of anti- and pro-apoptotic and BH3-only proteins. Under stress conditions the BH3-only proteins inhibit the anti-apoptotic proteins Bcl-2, Bcl-xl, Bcl-w and Mcl1 leading to mitochondrial outer membrane permeabilization (MOMP) through Bax and Bak oligomerization, activating the caspase cascade. Increased expression of anti-apoptotic proteins is a widely used strategy by cancer cells to increase the threshold for caspase activation and thereby prevention of apoptotic cell death^3^. Previously we already showed the importance of Bcl-2 family members in chondrosarcoma. Up regulation of Bcl-2 family members is an important mechanism causing chemo resistance, and combined inhibition of Bcl-2, Bcl-xl and Bcl-w with the BH3-mimetic ABT-737^4^ successfully induced sensitization of chondrosarcoma cell lines of all different subtypes to the chemotherapeutic agents doxorubicin and cisplatin^5–7^. Moreover, the anti-apoptotic protein Bcl-2 is up regulated in conventional chondrosarcoma, while expression of Bcl-xl and Bcl-w has not been studied^8^. In dedifferentiated and mesenchymal chondrosarcoma, Bcl-2 and Bcl-xl are highly expressed^7^. Unfortunately inhibition of Bcl-2 family members with the orally available derivative of ABT-737, ABT-263 resulted in a high toxicity rate (most significantly thrombocytopenia) in lymphoid malignancies and solid tumors^3^.

Our aim was to further unravel the role of the separate Bcl-2 family members, and to investigate whether selective inhibition of the separate Bcl-2 family members with possible lower toxicity could serve as an alternative to ABT-737. We evaluated the expression of the Bcl-2 family members Bcl-2, Bcl-xl and Bcl-w using immunohistochemistry on tissue microarrays containing 137 conventional chondrosarcomas. Since Bcl-2 and Bcl-xl were most abundantly expressed, we assessed the anti-tumoral effect of selective Bcl-2 inhibition using a novel BH3-mimetic, S55746^9^, which binds to the BH3-binding groove of Bcl-2, and selective Bcl-xl inhibition, using WEHI-539^10^ or its structurally related compound A-1155463^11^, in combination with chemotherapy. Studies were performed in vitro using a panel of chondrosarcoma cell lines of different histological subtypes, and in vivo in the orthotopic Swarm Rat Chondrosarcoma (SRC) model.

## Results

### Bcl-2 family members Bcl-2 and Bcl-xl are highly expressed in conventional chondrosarcoma

**Fig. 1:**
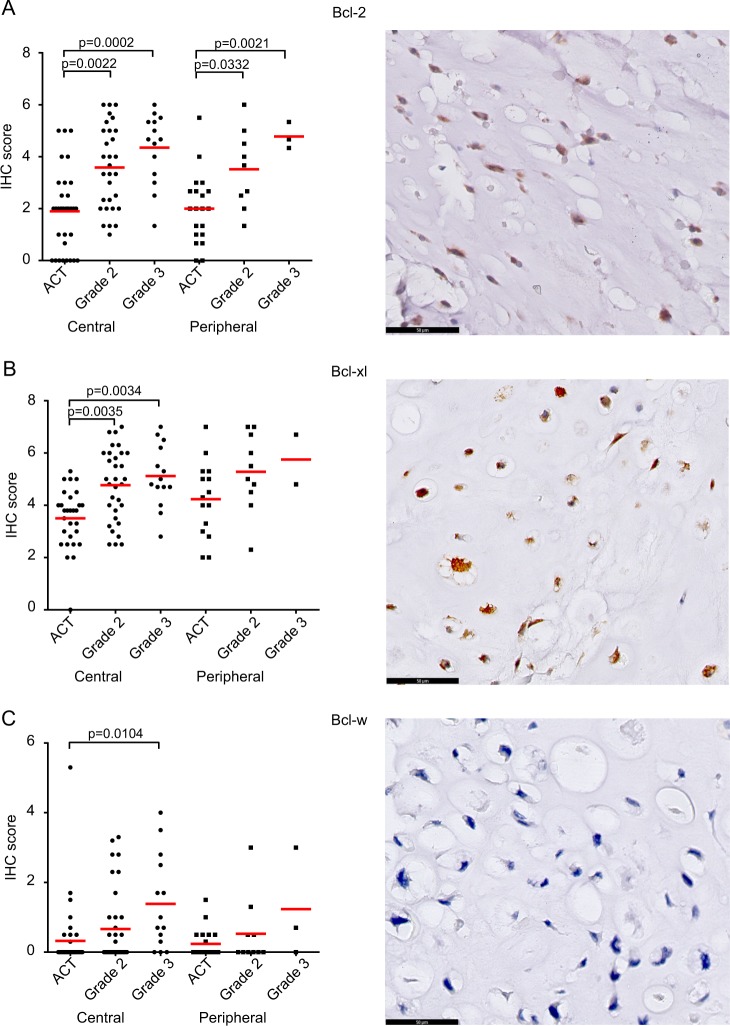
Bcl-2 and Bcl-xl are highly expressed in conventional chondrosarcoma. **a** Protein expression of Bcl-2 as detected by immunohistochemistry significantly increased with increasing histological grade, in peripheral as well as in central chondrosarcoma. **b** Bcl-xl is highly expressed in conventional chondrosarcoma and is increased in high grade (grade II and III) compared to ACT. **c** Expression of Bcl-w is low in conventional chondrosarcoma, but higher expression is observed in grade III chondrosarcoma compared to ACT. Each dot represents one tumor and mean value with standard deviation is shown for each group

Protein expression of Bcl-2, Bcl-xl and Bcl-w was determined in a panel of 137 conventional chondrosarcomas. Expression of Bcl-2 was variable and found in 100 out of 110 chondrosarcomas (27 were lost from the TMA) and correlated with histological grade in central as well as peripheral chondrosarcoma (Fig. 1a). Bcl-2 was significantly higher expressed in grade II (*P* = 0.0022) and grade III (*P* = 0.0002) central chondrosarcoma compared to ACTs. The same correlation with grade was observed in peripheral chondrosarcoma (ACT vs. grade II: *P* = 0.0332, ACT vs. grade III: *P* = 0.0021). Bcl-xl was expressed in 101 out of 102 chondrosarcomas (35 were lost from the TMA) and the extent and intensity significantly correlated with histological grade in central chondrosarcoma (ACT vs. grade II: *P* = 0.0035, ACT vs. grade III: *P* = 0.0034) (Fig. 1b). Expression of Bcl-w was found in 48 out of 120 chondrosarcomas and was higher in grade III central chondrosarcomas compared to ACTs (*P* = 0.0104) (Fig. 1c). No statistical differences were observed for Bcl-xl or Bcl-w in the peripheral chondrosarcomas, probably due to the smaller sample size. Examples of different ranges of Bcl-2 family member expression within one tumor are shown in supplementary figure 1A. Correlation between Bcl-2 and Bcl-xl was assessed using the Spearman correlation test showing a positive correlation (*r* = 0.5829, *P* < 0.0001) (supplementary figure 1B). The correlation was stronger in high grade chondrosarcomas (grade 2 and grade 3) (*r* = 0.6301, *P* < 0.0001) compared to low grade chondrosarcomas (*r* = 0.3393, *P* = 0.03461). For the central chondrosarcomas, *IDH* mutation status was known for 70 tumors, and there was no correlation between expression of Bcl-2 or Bcl-xl and *IDH1* or *2* mutation status (supplementary figure 1C). Taken together, Bcl-xl shows the highest expression in chondrosarcoma, followed by Bcl-2, suggesting that these two Bcl-2 family members are the most important, and were therefore selected for further functional validation.

### Chondrosarcoma cell lines show minimal sensitivity for selective Bcl-xl inhibition and are not sensitive for selective Bcl-2 inhibition

**Fig. 2:**
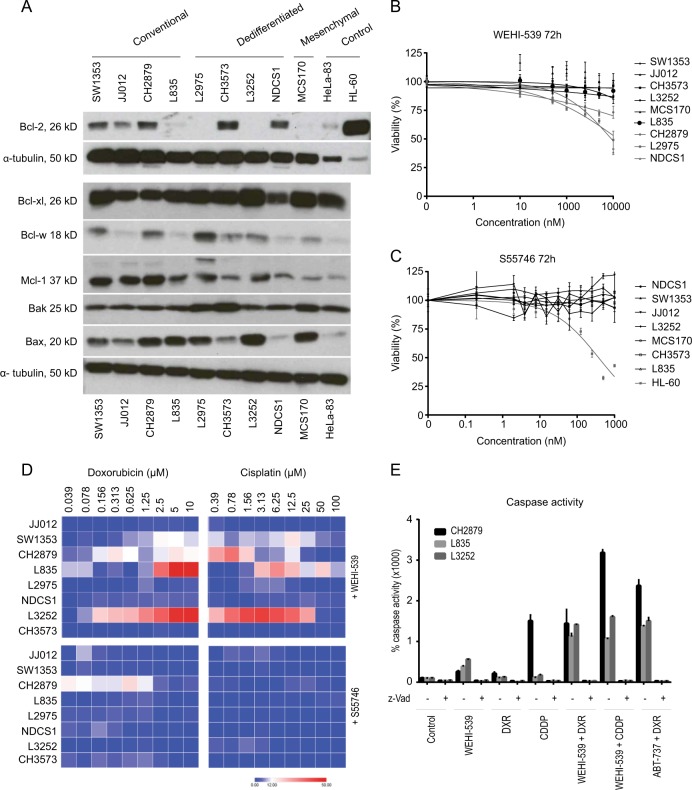
Bcl-xl can sensitize for chemotherapy in a subset of chondrosarcoma cell lines **a** Western blots showing expression of apoptotic regulators Bcl-2, Bcl-xl, Bcl-w, Mcl-1, Bak and Bax in chondrosarcoma cell lines. HeLa-83 and HL-60 cell lines are used as positive controls. Results for Bcl-2 where obtained in a separate experiment compared to the other protein expression analysis. **b** Dose response viability curves of chondrosarcoma cell lines after 72 h treatment with Bcl-xl inhibitor WEHI-539. Chondrosarcoma cell lines NDCS1, L2975 and CH2879 show minimal sensitivity towards treatment with Bcl-xl inhibitor WEHI-539. **c** Dose response viability curves of chondrosarcoma cell lines after 72 h treatment with Bcl-2 inhibitor S55746 show that chondrosarcoma cell lines are not sensitive for S55746, as compared to the positive control HL-60. **d** Excess over Bliss percentages of chondrosarcoma cell lines treated with doxorubicin or cisplatin in combination with WEHI-539 or S55746 for 72 h. Values were obtained by addressing the viability using presto blue assays. Chondrosarcoma cell lines L835 and L3252 show increased sensitivity towards doxorubicin and cisplatin when co-treated with WEHI-539. In addition CH2879 and SW1353 show increased sensitivity towards cisplatin when treated in combination with WEHI-539. A small increase in sensitivity is observed in CH2879 when S55746 is combined with doxorubicin. **e** Caspase 3/7 activity of CH2879, L835 and L3252 cell lines after treatment with WEHI-539 (0.5 or 5 µM), Doxorubicin (1 µM), Cisplatin (10 µM) or a combination. As a positive control cells have been treated with ABT-737 and doxorubicin. Addition of pan-caspase inhibitor Z-vad showed that measured activity was specific. (DXR = doxorubicin, CDDP = cisplatin)

Protein expression analysis of apoptotic family members Bcl-2, Bcl-xl, Bcl-w, Mcl-1, Bak and Bax revealed that Bcl-xl was highly expressed in all nine chondrosarcoma cell lines, while Bcl-2 expression was variable (Fig. 2a). Bcl-2 was expressed in three conventional and two dedifferentiated chondrosarcoma cell lines and is not correlated to *IDH* mutation status^12^. Bcl-w expression was variable and was highest in L2975. Bak and Bax are both expressed in all chondrosarcoma cell lines although expression of Bax is variable. Interestingly the same trend is observed as shown with immunohistochemical staining; Bcl-xl is highly expressed in all cell lines. Inhibition of Bcl-xl using WEHI-539 resulted in a reduction in cell viability in a subset of chondrosarcoma cell lines (Fig. 2b). The most sensitive chondrosarcoma cell lines were conventional chondrosarcoma cell line CH2879 and dedifferentiated chondrosarcoma cell lines L2975 and NDCS1. In contrast, no sensitivity for Bcl-2 inhibition using S55746 was observed in any of the chondrosarcoma cell lines tested, while the positive control cell line HL-60 showed a dose dependent decrease in viability (Fig. 2c).

### Selective Bcl-xl but not Bcl-2 inhibition can sensitize for conventional chemotherapy in a subset of chondrosarcoma cell lines

Inhibition of Bcl-xl with WEHI-539 led to increased sensitivity to doxorubicin or cisplatin treatment in a subset of chondrosarcoma cell lines, while combination treatment with Bcl-2 inhibitor S55746 and chemotherapy did not result in a difference in sensitivity compared to single treatment (Fig. 2d., supplementary figure 2 and supplementary figure 3). Combination treatments with chemotherapy were performed with either 1 µM S55746 or 0.5 µM WEHI-539 for the sensitive cell lines (CH2879, L2975 and NDCS1) or 5 µM WEHI-539 for the more resistant cell lines (JJ012, SW1353, L835, L3252 and CH3573). Bliss independence values were calculated and showed that the most chemo resistant cell lines L835 and L3252 could be sensitized for doxorubicin and cisplatin by inhibition of Bcl-xl using WEHI-539. In addition, CH2879 and SW1353 showed an increase in sensitivity towards chemotherapy after Bcl-xl inhibition (Fig. 2d and supplementary figure 2). Inhibition of Bcl-2 using S55746 in combination with doxorubicin or cisplatin only showed a small effect in CH2879 cell line, while in all other cell lines no difference was observed (Fig. 2d and supplementary figure 3). In addition, an increase in caspase activation was observed when CH2879, L835 or L3252 was treated with WEHI-539 (0.5 µM WEHI-539 CH2879, 5 µM L835 and L3252) and chemotherapy compared to single treatment (Fig. 2e). Single treatment with WEHI-539 or doxorubicin or cisplatin resulted in a 200–500% increase in caspase dependent apoptosis compared to the 100% control in all cell lines. The only exception was CH2879 treated with cisplatin which resulted in a 1700% increase. Combination treatment resulted in an increase of 1500–3500% compared to the control indicating that the sensitizing effect we observe is depending on an increase in caspase dependent apoptosis.

### Inhibition of Bcl-xl but not Bcl-2 results in a decrease in tumor growth in a Swarm Rat chondrosarcoma model

**Fig. 3:**
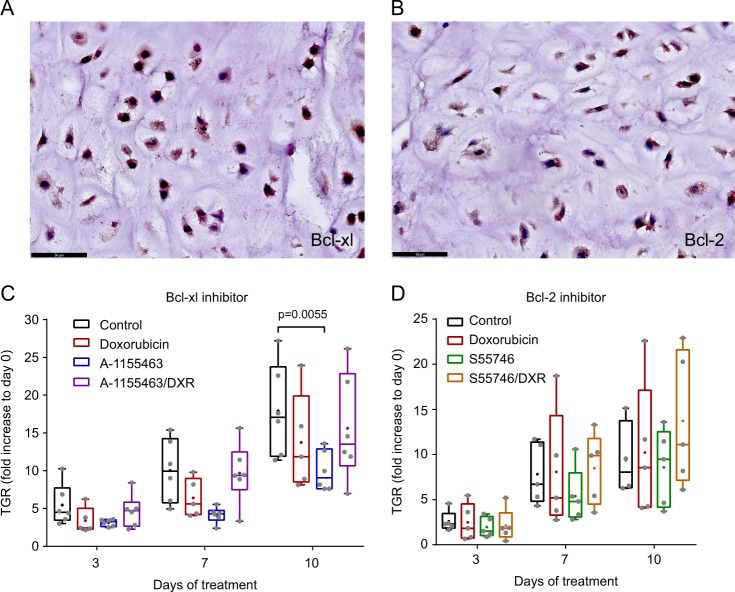
Bcl-xl inhibition is more effective in Swarm rat chondrosarcoma compared to Bcl-2 inhibition. High protein expression of Bcl-xl (**a**) and Bcl-2 (**b**) in the SRC tissue determined by immunohistochemistry. **c** Tumor growth rate (TGR) of tumors orthotopically implanted in rats on day 3, 7 and 10 compared to day 0. Rats were treated with doxorubicin, A-1155463 or a combination. Treatment with A-1155463 resulted in a significant decrease in TGR compared to control mice. **d** TGR of tumors orthotopically implanted in rats on day 3, 7 and 10 compared to day 0. Rats were treated with doxorubicin, S55746 or a combination. No statistical difference is observed between the different treatment groups

Expression of Bcl-2 and Bcl-xl was determined in Swarm rat chondrosarcoma to evaluate their suitability as a representative model to study Bcl-2 family member inhibitors. Figure 3a, b shows that Bcl-xl, as well as Bcl-2 are highly expressed in these tumors as determined by immunohistochemistry, mimicking the human situation. The effect of Bcl-xl inhibition was studied using A-1155463, a structurally related compound to WEHI-539, shown to cause less toxicity in vivo. A concentration of 5 mg/kg was used, since this had shown on target activity before in mice^11^. Inhibition of Bcl-xl showed a significant (P = 0.0055) decrease in tumor growth compared to control or doxorubicin treated rats (Fig. 3c), however no difference was observed when A-115463 was combined with doxorubicin, indicating that single Bcl-xl inhibition is more effective. Since Bcl-2 inhibitor S55746 was never tested in rats before a dose escalation study was performed (25, 50, 100 and 150 mg/kg). A biweekly administration of 50 mg/kg S55746 resulted in the smallest increase in tumor volume (1218 ± 403 mm^3^ in treated group vs. 2033 ± 647 mm^3^ in the PBS control group at day 14) and was selected for further experiments (supplementary figure 4). Inhibition of Bcl-2 did not lead to a significant reduction in tumor growth as a single agent nor in combination with doxorubicin. (Fig. 3d).

## Discussion

Bcl-2 family members have been investigated as therapeutic targets in a large variety of solid tumors as well as in chondrosarcoma. Previously we showed that chondrosarcoma cell lines of all different subtypes can be sensitized to chemotherapy by inhibiting Bcl-2 family members using ABT-737^5–7^. In this study we investigated whether single Bcl-2 or Bcl-xl inhibition could be an alternative therapeutic strategy for patients with chondrosarcoma, since inhibition of Bcl-2, Bcl-xl and Bcl-w using ABT-263, showed toxicity problems when used in the clinic for other malignancies^3^. Bcl-2 and especially Bcl-xl were highly expressed in chondrosarcoma tissue samples, which correlated with an increased histological grade. Bcl-w was also expressed but not as abundantly, and also correlated with histological grade. In addition a positive correlation between Bcl-2 and Bcl-xl expression was observed, especially in high grade chondrosarcomas. These results are in line with previously published smaller studies, conducted by us as well as others, in which Bcl-2 and Bcl-xl also showed an increased expression with increasing histological grade^8,13–15^. Selective Bcl-xl inhibition with WEHI-539 was effective at relatively high doses in a subset of chondrosarcoma cell lines, which did not correlate with expression of Bcl-2 or Bcl-xl. These doses were comparable with concentrations obtained in osteosarcoma cell lines^16^. Our in vivo data confirm that tumor growth can be slowed down by selective inhibition of Bcl-xl by A-1155463, a structurally related Bcl-xl inhibitor showing less toxicity in vivo^11^. When the combination with chemotherapy was investigated the two cell lines that were most resistant to doxorubicin or cisplatin (L835 and L3252) could be sensitized for these agents when combined with WEHI-539. In addition, we confirmed that the combination treatment induced apoptosis. Interestingly those two cell lines did not show Bcl-2 and low Bcl-w expression. In the SRC model, the combination of selective Bcl-xl inhibition and doxorubicin did not show a sensitizing effect, and thus did not confirm the advantage of combination treatment suggested by the in vitro data. However, while the cell lines show a heterogeneous response with respect to chemo-sensitization, with a preference for chemo-resistant cell lines with high Bcl-xl and low Bcl-2 and Bcl-w expression, the SRC model represents only a subset of the chondrosarcomas.

Although half of the chondrosarcoma cell lines showed Bcl-2 expression, none of the cell lines was sensitive for selective Bcl-2 inhibition with S55746 and only one cell line (CH2879) showed a small increase in sensitivity for doxorubicin when treated in combination with the Bcl-2 inhibitor. In line with this, in the orthotopic Swarm rat chondrosarcoma model, S55746 also showed no beneficial effect regarding inhibition of tumor growth. These data indicate that Bcl-2 is not essential for the survival of chondrosarcoma cells, and its function might be taken over by other apoptosis proteins. Bcl-xl is essential only in a subset of chondrosarcoma cell lines, and also seems important in the rat chondrosarcoma model. This means that the previously observed effects of combined inhibition of Bcl-2, Bcl-xl and Bcl-w using ABT-737 cannot be substituted by selective inhibition of Bcl-2 alone, not even in the cell lines that highly express Bcl-2. This can be caused by the high Bcl-xl expression observed in all chondrosarcoma cell lines, which has been reported previously as a possible cause of resistance to selective Bcl-2 inhibition^17,18^. In the majority of the high grade chondrosarcomas high Bcl-2 and Bcl-xl expression is observed, which might suggest that selective Bcl-2 inhibition could be problematic due to high Bcl-xl expression or vice versa. However, high Bcl-2 expression does not confer resistance towards Bcl-xl inhibition as shown in a study by Punnoose et al. in multiple myeloma pointing towards Bcl-xl as a more promising target in tumors expressing both Bcl-2 and Bcl-xl^18^. No difference in sensitivity for Bcl-2 or Bcl-xl inhibition was observed between *IDH1* or −*2* mutant cell lines and wild type cells. This is different from acute myeloid leukemia in which Bcl-2 inhibition was identified as synthetic lethal to elevated levels of the oncometabolite D-2HG caused by mutations in *IDH1* or *IDH2*. The authors propose a model in which D-2HG inhibits the activity of cytochrome c oxidase (COX) in the mitochondrial electron transport chain, which lowers the threshold to trigger apoptosis after Bcl-2 inhibition^12^. In addition, *IDH1* mutant gliomas were more sensitive for Bcl-xl inhibition, which was shown to be dependent on D-2HG, which lowers Mcl-1 expression in mutant gliomas compared to wild type gliomas thereby increasing sensitivity for Bcl-xl inhibition^19^. The different results in these different tumor types sharing a mutation in *IDH* emphasize that these mutations may have a tissue specific effect which hampers the development of a common therapy for *IDH* mutant tumors.

The importance of Bcl-xl over Bcl-2 regarding chemo resistance is in line with results found in several other solid tumors. One study testing ABT-263, Bcl-2 and Bcl-xl selective inhibitors in a panel of breast cancer, non-small lung cancer and ovarian cancer cell lines, showed that only ABT-263 and the Bcl-xl selective inhibitors where able to induce sensitization to docetaxel, indicating that Bcl-xl is the most important protein causing chemo resistance^20^. Also in osteosarcoma cell lines a selective Bcl-2 inhibitor could not sensitize for doxorubicin, whereas WEHI-539 treatment resulted in a synergistic effect^16^. A correlation between Bcl-xl and chemo resistance has been shown before in a study in which they found a strong correlation between Bcl-xl mRNA expression and the sensitivity of 60 cell lines towards 122 standard chemotherapy agents^21^. No clinical trials have been performed using selective Bcl-xl inhibitors, however the Bcl-xl inhibitor A-1155463 that we tested in vivo and its orally available related compound A-1331852 seem promising in pre-clinical studies in rodents^11,20^. Furthermore a study by Leverson et al shows that in rodents higher concentrations of Bcl-xl inhibitors in combination with docetaxel can be administered before thrombocytopenia becomes dose limiting, in comparison with combination therapy using ABT-263, in which, because of Bcl-2 inhibition, suppression of granulopoiesis and neutropenia becomes dose limiting at much lower concentrations^20^. Although a clear biomarker to predict sensitivity still needs to be identified, we show that among the Bcl-2 family members, Bcl-xl is most important for chondrosarcoma survival. Further research is needed to validate whether single or combination treatment with chemotherapy will be beneficial for chondrosarcoma patients.

## Material and methods

### Compounds

The selective Bcl-2 inhibitor S55746 was kindly provided and developed by Servier (Suresnes, France). For in vitro experiments S55746 was dissolved in phosphate buffered saline (PBS). ABT-737 (S1002, Selleckchem, Houston, TX, USA), WEHI-539 (ApexBio Technology, Houston, TX, USA) and Z-vad-FMK^22^ (550377, BD biosciences, San Jose, CA, USA) were dissolved in DMSO and stored at −20 °C. The animal experiments were performed with freshly dissolved S55746 according to the manufacturer’s indications at 30 mg/mL in a solution of 40% polyethylene glycol (Sigma-Aldrich, St Louis, MO, USA), 10% ethanol (Sigma-Aldrich) and 50% sterile water for injection (B. Braun, Melsungen, Germany). A-1155463 (S7800, Selleckchem, Houston TX USA) was dissolved in 5% DMSO, 10% ethanol, 20% PEG 35 and 65% sterile water as recommended by the manufacturers. Doxorubicin (2 mg/ml in a 0.9% NaCl solution) and cisplatin (1 mg/ml in a 0.9% NaCl solution) were obtained from the in house hospital pharmacy from the Leiden University Medical Centre for in vitro experiments or Centre Léon Bérard for in vivo experiments.

### Cell culture

Chondrosarcoma cell lines JJ012^23^, SW1353 (ATCC), CH2879^24^, L2975^25^ and NDCS1^26^ and control cell lines HeLa (ATCC) and HL-60 (ATCC) were cultured in RPMI-1640 (Gibco, Invitrogen Life-Technologies, Scotland, UK) containing 10% Fetal Calf Serum (Gibco, Invitrogen Life-Technologies, Scotland, UK). Chondrosarcoma cell lines CH3573^27^, L3252^25^ and L835^25^ were cultured in RPMI-1640 containing 20% Fetal Calf Serum. Mesenchymal chondrosarcoma cell line MCS170^5^ was cultured in IMDM medium (Gibco, Invitrogen Life-Technologies, Scotland, UK) supplemented with 15% Fetal Calf Serum. JJ012 and L835 cell lines are known to contain an *IDH1* mutation, while SW1353 and L2975 show a mutation in *IDH2*. All other cell lines are *IDH1* and *IDH2* wild type^28^. Cell lines were cultured in a humidified incubator (5% CO_2_) at 37 °C. Before and after completion of the experiments cell identity was confirmed using the Cell ID Gene Print 10 system (Promega Benelux BV, Leiden, The Netherlands). Mycoplasma negativity was confirmed on a regular basis.

### Swarm rat chondrosarcoma model

All animal experiments were performed in accordance with European and French regulations and protocols were authorized by the animal ethical evaluation committee C2EA-UCBL55, (protocol number: DR2014-49). All experiments were conducted in the pathogen-free animal facilities SCAR (Faculté de Médecine Rockefeller, Université Claude Bernard Lyon 1, Lyon, France) at the Rockefeller Medicine faculty (Agreement # A 69 388 10 01). The Swarm rat chondrosarcoma model (SRC) is a transplantable in vivo model which has been described previously^29^. It mimics the aggressiveness and chemo-resistance observed in human chondrosarcoma as well as histological features and it is classified as a grade II chondrosarcoma.^30^. Tumor fragments of 10 mm^3^ were grafted on the right posterior tibia of 1 month old Spraque-Dawley rats. Before transplantation rats underwent periosteal abrasion. Palpable tumors were observed after approximately 10 days upon which rats were randomly divided into treatment groups consisting of 5–6 rats in each group. Treatment was given for a period of 10–14 days until tumors reached 2500 mm^3^. Treatment response was evaluated by monitoring tumor growth by regular visual inspection and tumor dimensions were measured every 2–3 days (no blinding was done). Calculation of the tumor volume was performed using the following formula: Volume = (longest tumor diameter × (shortest tumor diameter)^2^)/2. Rats were sacrificed when tumors reached 2500 mm^3^ or after completion of the treatment period. Tumors were harvested and fixed in 10% formalin for Immunohistochemical analysis. The following treatments were given twice a week either by IP administration or per oral gavage (S55746): S55746 (25, 50, 100 or 150 mg/kg), A-1155463 (5 mg/kg;) and/or Doxorubicin (1 mg/kg).

### Immunohistochemical analysis

Previously constructed tissue micro arrays (TMAs) containing 137 conventional chondrosarcomas (92 central of which 42 grade I, 36 grade II, 14 grade III and 45 peripheral including 31 grade I, 11 grade II, 3 grade III)^31^ were stained for Bcl-2 (Dako, clone 124, Agilent Technologies, Santa Clara, CA, USA), Bcl-xl (Cell signaling, clone 54H6, Danvers MA, USA) and Bcl-w (Abcam, clone 6C1) protein expression. Tonsil was used as a positive control for Bcl-2, prostate for Bcl-xl and cerebellum for Bcl-w. Cytoplasmic staining was scored separately by two observers (JVMGB, YDJ) using a scoring system assessing intensity (0 = no, 1 = weak, 2 = moderate, 3 = strong) and percentage of staining (0 = no, 1 = 1–24%, 2 = 25–49%, 3 = 50–74%, 4 = 75–100%)^7^ and discrepancies were discussed to reach consensus. *IDH* mutation status was determined for 70 central chondrosarcomas in a previous study^32^, and correlation towards Bcl-2, Bcl-xl and Bcl-w immunohistochemistry expression was assessed. The protocol was validated and approved by the medical ethical evaluation committee (protocol number: B17.020). Immunohistochemical analysis of rat chondrosarcomas was performed to determine Bcl-2 and Bcl-xl expression using the following primary antibodies: anti-Bcl-2 (polyclonal rabbit; Bio Vision, Milpitas, USA) and anti-Bcl-xl (clone 54H6, Cell Signaling, Danvers, USA). Goat anti-rabbit antibody (AI-1000; Vector Lab, Burlingame, CA, USA; dilution 1:100) was used as a secondary antibody and detection was performed using avidin-biotin complex and visualization with DAB peroxidase. (VECTASTAIN Elite ABC Reagent, ImmPACT reagent; Vector Lab).

### Viability assay

Chondrosarcoma cells were seeded in 96 well plates (3000–20000 cells depending on the cell line) and were treated with increasing concentrations of S55746 (0–1000 nM) or WEHi-547(0–10 uM) for 72 h. HL-60 cells were used as positive control cells. Combination treatments with doxorubicin or cisplatin where performed with either 0.5 µM of S55746 or 5 or 0.5 µM of WEHI-547. Presto blue reagent (Invitrogen, Life-Technologies, Scotland, UK) was used as described by the manufacturer to measure cell viability. Fluorescence was measured at 590 nm as a read out for cell viability on a victor3V mutilabel reader.(Perkin Elmer, the Netherlands). Three technical replicates were included in each experiment and assays were repeated 2–3 times.

### Caspase assay

The caspase-glo 3/7 assay from promega (Madison, USA) was used to measure apoptosis induction. Chondrosarcoma cell lines CH2879, L835 and L3252 were plated in white walled 96 well plates (Corning B.V. Life Sciences, Amsterdam, the Netherlands). After overnight attachment cells were treated for 24 h with either WEHI-539, doxorubicin, cisplatin or a combination. To measure caspase 3/7 activity the substrate was added in a 1:1 dilution with medium and incubated for half an hour at room temperature. Cells treated with a combination of ABT-737 and doxorubicin were included as a positive control. As an additional control treatment with pan-caspase inhibitor Z-VAD-FMK was performed. Luminescence was measured with a victor V3 multilabel reader (Perkin Elmer, Netherlands). Each experiment was performed in triplicate and the assay was repeated three times.

### Western blotting

Western blotting was performed for Bcl-xl (Cell signaling, clone 54H6), Bcl-w (Abcam, clone 6C1), Mcl-1, Bak (Cell signaling, clone D4E4) and bax (Cell signaling, clone D2E11). Western blotting for Bcl-2 was performed with two different clones (Cell signaling, clone D55G8 and clone 50E3) of which clone D55G8 was proven to give the most reliable results and was therefore chosen to be used for experiments. Cells were lysed using hot-SDS buffer (1% SDS, 10 mM Tris/EDTA with complete inhibitor and phosSTOP) and 20 µg protein was loaded for each sample. Otherwise the procedure was performed as previously described^33^. α-tubulin (clone DM1A, Sigma-Aldrich Chemie B.V. Zwijndrecht, the Netherlands) expression was determined as a loading control. furthermore

### Statistical analysis

Results were analyzed using a 2way ANOVA followed by Turkey’s multiple comparisons test using GraphPad Prism v6. software (GraphPad Software, Inc., La Jolla, CA, USA). Correlation tests were performed using the Spearman correlation test in graphpad Prism v6. Results are given as mean ± SD and results with *p* < 0.05 were considered significant. The Bliss independence model was used to evaluate synergy between treatment combinations^34^. Using the formula C = A + B − A × B, in which C represents combined response and A and B the two single compounds, predictions were made to assess synergy^35^. The heatmap figure was generated using the MORPHEUS online tool.

## Electronic supplementary material

Supplementary figure 1

Supplementary figure 2

Supplementary figure 3

Supplementary figure 4
