# Supplementary material for: Bcl-xl as the most promising Bcl-2 family member in targeted treatment of chondrosarcoma
Source: Oncogenesis. 2018 Sep 21;7(9):74. doi: 10.1038/s41389-018-0084-0 (PMC6155044; doi:10.1038/s41389-018-0084-0)
Supplement: Supplementary file 1 — Supplementary figure 1 [file 41389_2018_84_MOESM1_ESM.docx]

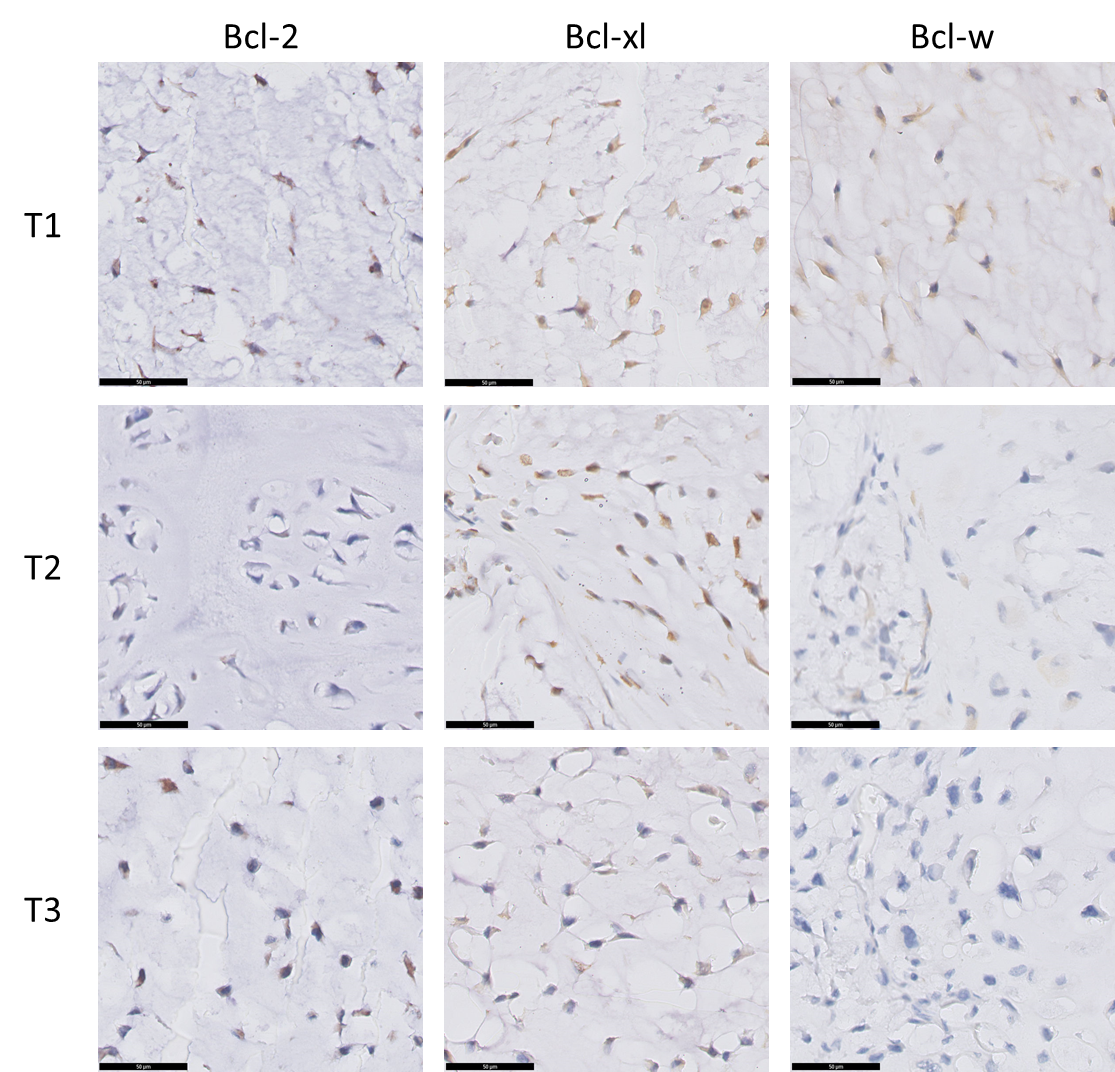


C

B

A

Supplementary figure 1.

**A**. Immunohistochemical staining results of three different cases of high grade chondrosarcoma tumor samples showing different staining patterns for Bcl-2, Bcl-xl and Bcl-w. **B**. A positive correlation is observed between Bcl-2 and Bcl-xl expression in chondrosarcoma patient tissue samples (r=0.5829, P<0.0001). Blue = Grade I , Green = Grade II, Red = Grade III. Higher grade chondrosarcomas show a stronger correlation (r=0.6301, P<0.0001) compared to low grade chondrosarcomas (r=0.03393, P=0.03461) Correlation is determined using the spearman correlation test. **C.** Bcl-2 and Bcl-xl expression are not correlated towards *IDH* mutation status
