# Supplementary material for: Bcl-xl as the most promising Bcl-2 family member in targeted treatment of chondrosarcoma
Source: Oncogenesis. 2018 Sep 21;7(9):74. doi: 10.1038/s41389-018-0084-0 (PMC6155044; doi:10.1038/s41389-018-0084-0)
Supplement: Supplementary file 2 — Supplementary figure 2 [file 41389_2018_84_MOESM2_ESM.docx]

Supplementary figure 2.

Inhibition of Bcl-xl with WEHI-539 results in an increased sensitivity for doxorubicin (DXR) and cisplatin (CDDP) in L835 and L3252 cell lines. In addition SW1353 and CH2879 show a small increase in sensitivity for cisplatin.
