# Supplementary material for: Bcl-xl as the most promising Bcl-2 family member in targeted treatment of chondrosarcoma
Source: Oncogenesis. 2018 Sep 21;7(9):74. doi: 10.1038/s41389-018-0084-0 (PMC6155044; doi:10.1038/s41389-018-0084-0)
Supplement: Supplementary file 3 — Supplementary figure 3 [file 41389_2018_84_MOESM3_ESM.docx]

Supplementary figure 3.

Inhibition of Bcl-2 with S55746 does not result in an increased sensitivity for doxorubicin (DXR) or cisplatin (CDDP) in all chondrosarcoma cell lines. Only CH2879 shows a small increase in sensitivity for doxorubicin.
