# Supplementary material for: Bcl-xl as the most promising Bcl-2 family member in targeted treatment of chondrosarcoma
Source: Oncogenesis. 2018 Sep 21;7(9):74. doi: 10.1038/s41389-018-0084-0 (PMC6155044; doi:10.1038/s41389-018-0084-0)
Supplement: Supplementary file 4 — Supplementary figure 4 [file 41389_2018_84_MOESM4_ESM.docx]

Supplementary figure 4. Dose escalation study of S55476 in Swarm Rat chondrosarcoma model. (n=3-4 Rats/group).
